# Demand for Modern Family Planning among Married Women Living with HIV in Western Ethiopia

**DOI:** 10.1371/journal.pone.0113008

**Published:** 2014-11-12

**Authors:** Tesfaye Regassa Feyissa, Alemu Sufa Melka

**Affiliations:** College of Medical and Health sciences, Wollega University, Nekemte, Oromia, Ethiopia; Harvard Medical School, United States of America

## Abstract

**Introduction:**

People living with HIV (PLHIV) have diverse family planning (FP) needs. Little is reported on FP needs among women living with HIV in Ethiopia. Thus, the objective of the study was to assess the demand for modern FP among married women living with HIV in western Ethiopia.

**Methods:**

A facility-based cross-sectional survey was conducted on 401 married women living with HIV selected from Nekemte Referral Hospital and Health Center, Nekemte, Oromia, Ethiopia. Convenience sampling of every other eligible patient was used to recruit respondents. Data were collected using a pretested, structured questionnaire. We first calculated frequency and percentage of unmet need, met need and total demand by each explanatory variable, and performed chi-squared testing to assess for differences in groups. We then fitted logistic regression models to identify correlates of unmet need for modern FP at 95% CL.

**Results:**

The proportion of respondents with met need for modern FP among married women living with HIV was 61.6% (30.7% for spacing and 30.9% for limiting). Demand for family planning was reported in 77.0% (38.2% for spacing and 38.8% for limiting), making unmet need for modern FP prevalent in 15.4% (7.5% for spacing and 7.9% for limiting). Whereas age 25–34 years [adjusted odds ratio (AOR) (95% confidence interval (CI))  = .397 (.204–.771)] was protective against unmet need for modern FP, not having knowledge of MTCT [AOR (95% CI)  = 2.531 (1.689–9.290)] and not discussing FP with a partner [AOR (95% CI)  = 3.616(1.869–6.996)] were associated with increased odds of unmet need for modern FP.

**Conclusions:**

There is high unmet need for modern FP in HIV-positive married women in western Ethiopia. Health care providers and program managers at a local and international level should work to satisfy the unmet need for modern family planning.

## Introduction

Over 50% of the 33.3 million HIV-positive people are women within the reproductive age [1]. Women account for 59% of the people Living with HIV (PLHIV) in Ethiopia [2]. Although HIV prevalence and fertility rates in Ethiopia are among the highest in the world, little is known about how HIV infection affects the met and unmet need for modern family planning [2]. Prevention of unintended pregnancies among HIV infected women is among the four key pillars for comprehensive prevention of mother to child transmission (PMTCT). This could be achieved with the use of contraception[3], [4].

HIV-infected women have a heightened need for guidance from health care providers prior to an attempt for pregnancy because of the potential risks of transmission to the partner while trying to conceive and to the infant during pregnancy, delivery and breast feeding [5], [6]. Providing HIV-infected women with HIV care, opportunities to plan and space pregnancies, and quality pregnancy care including PMTCT services improve health outcomes for both mothers and infants [7]. Effective programs to prevent perinatal HIV transmission would, if accessed by all women in need, prevent approximately 300,000 HIV transmissions annually [8], [9].

However, there is considerable evidence that HIV infected women have high rates of unmet need for contraception [2], [10], [11], [12], [13]. Previous studies in Ethiopia have focused on demand and unmet need for family planning among married women in general population [2], [14]. We conducted a survey of women at an HIV clinic in Ethiopia to learn about unmet need and demand for modern family planning among married women living with HIV. The objective of the study was to assess demand for modern family planning and extent of unmet need for family planning among women living with HIV in Western Ethiopia. We intend that results of this study could assist policy makers, health managers and implementers to design appropriate strategies to address family planning needs for people living with HIV in this setting.

## Materials and Methods

### Study design and setting

We performed a facility-based, cross-sectional survey among married/in union women living with HIV in western Ethiopia between February and March 2014**.** The study was undertaken in Nekemte Referral Hospital and Health Center. Nekemte, capital of East Wollega zone, is located 331 kilometers from Addis Ababa to the west. The total estimated number of women living with HIV in Ethiopia was 448,865 of whom 254,931 were women meeting criteria for antiretroviral therapy (ART) in 2013. There were 34,524 HIV positive pregnant women needing PMTCT in 2013 [15].

### Study population and participant sampling

The study population was married/in union women of reproductive age living with HIV who had at least one visit to the selected health institutions during the study period for ART services. Women living with HIV and aged 15–49 years were eligible for participation in the study. Further inclusion criteria were that they were willing to discuss certain aspects of their contraception use, and have basic information abstracted from their medical records. Clients who were severely ill were excluded from the study because it was considered they could not give necessary information. The estimated number of married/in union women attending the two ART clinics was 950. Convenience sampling was employed to select study participants using registers of patients attending each institution. Every second married/in union women was enrolled into the study.

### Study questionnaire and procedures

The questionnaire was derived from related questions in the Demographic Health Survey. The questionnaire for the survey was initially developed in English, translated to local language, Afan Oromo, and checked for consistency by back translation to English by two different individuals. The questionnaire was also pretested on 5% of the total sample size in the ART units of Sibu Sire health center and Gimbi Hospital. The questionnaire was then assessed for its clarity and completeness. Some skip patterns were corrected, questions difficult to ask were rephrased and the consent form was modified. Four nurses working in the ART units of each health institution administered the questionnaire after a two-day study training. Data were entered into Epi-Info 6.04 then transferred to SPSS version 20.0 for analysis.

### Statistical analyses

We first calculated frequency and percentage of unmet need, met need and total demand by each explanatory variable, and performed chi-squared testing to assess for differences between groups. We then fitted logistic regression models to identify correlates of unmet need for modern family planning (see definition below). Bivariate analysis was performed between unmet need and each of the independent variables separately. To control for possible confounding variables, multivariable logistic regression was done. All variables which had association in bivariate analysis (*P*-value <0.05) were included in the multivariate model. The strength of association between unmet need for modern family planning and independent variables was expressed in odds ratio (OR) through a 95% confidence interval.

Our primary outcome of interest was unmet need for modern family planning. Unmet need for modern family planning was defined as any sexually active woman with HIV who said they wanted to stop childbearing, delay their next birth by at least two years, or were unsure when to have a child but were not using a modern method of family planning. Family planning refers to the use of modern contraceptives or natural techniques to limit or space pregnancies. Modern family planning methods included the pill, female and male sterilization, IUD, injectables, implants, male and female condom, and diaphragm. Pregnant or amenorrheic women were also considered to have an unmet need if their current or most recent pregnancy was unwanted or mistimed, and they were not using a method of family planning. For amenorrheic women, only those who gave birth in the last six months were included [16]. Pregnant and postpartum women may not have had or known they had HIV at the time of that pregnancy which may have slight difference on level of unmet need. Four pregnant or amenorrheic women reported contraceptive failure (one who wanted to limit and three who want space). They were excluded from the analysis.

The explanatory variables included in the model were socio-demographic factors (income, education, religion, occupation, and functional radio and/or TV), number of children, time since HIV positive diagnosis, recent CD4, duration on ART, current pregnancy status of respondent, current fertility desire, and discussion on family planning, fecundity. Duration of HIV diagnosis, CD4 counts and length of time on ART were abstracted from medical records.

Women who never used contraception, and not had a birth in past five years, reported they “can't get pregnant” or have had a hysterectomy were considered infecund for this study. We defined satisfied demand as the percentage of met need to total demand. Knowledge of MTCT was defined as knowing of transmission from mother to child during any of the following; a) pregnancy b) labour and delivery or c) breast feeding. Pregnancy intendedness was categorized as a) wanted the pregnancy at that time (want then), b) wanted later (mistimed) or c) did not want any child at all (unwanted). Respondents were asked about the frequency of discussion on family planning with a partner and the response was categorized as a) never discussed, b) rarely discussed, or c) discussed often.

### Ethical considerations

Ethical approval was obtained from Ethical review committee of Wollega University, Nekemte, Ethiopia. A formal letter for permission and support was written to health institutions. The purpose of the study was clearly explained to concerned bodies. In order to keep confidentiality of patient information, only those personnel who were working in the ART unit were involved in data collection. The purpose and process of the study was explained to all participants. Written informed consent was obtained from all study participants. They were informed that their participation was voluntary and they were free whether or not to participate, or to withdraw at any time and for any reasons without any penalty either personnel or affecting their future medical care. For study participants aged less than 18, we received written informed consent from the respondents themselves because respondents were married and mature minor. The consent procedure was approved by the ethics committee for all including aged less than 18 years.

## Results

### Socio-demographic characteristics

We approached 403 study participants, of whom 401 responded to the questionnaire for a response rate of 99.5%. Sixty percent of respondents were in the age group of 25–34 years with a mean age of 29.4 years (SD±5.4 years, Table 1). A majority of the study participants were residing in an urban area (81.3%).The proportion of women with no formal education was 42.6%. Seventy five percent of the respondents earned monthly family income of less than 1275 Ethiopian birr (ETB) (about $67 USD).

**Table 1:** Characteristics of married women living with HIV in western Ethiopia, March 2014.

Variables	Categories	frequency (n = 401)	%
Age	15–24	69	17.2
	25–34	242	60.3
	35–49	90	22.4
Mean age (±SD)		29.4 years (± 5.4 years)	
Education level	No formal education	171	42.6
	Primary education	131	32.7
	Secondary and above education	99	24.7
Education level of partner	No formal education	91	22.7
	Primary education	124	30.9
	Secondary and above education	186	46.4
Religion	Protestant Christian	163	40.6
	Orthodox Christian	186	46.4
	Muslim	52	13.0
Occupation	Daily labourer	88	21.9
	Employed- skilled	182	45.4
	Housewife	73	18.2
	Farmer	58	14.5
Residence	Urban	326	81.3
	Rural	75	18.7
Monthly income quintile	Lowest	95	23.7
	Second	82	20.4
	Middle	77	19.2
	Fourth	73	18.2
	Highest	74	18.5
Family size	2 or less	45	11.2
	3–4	195	48.6
	Greater than four	161	40.1
Number of live children	No living child	47	11.7
	1–2	218	54.4
	>2	136	33.9
recent CD_4_ count	<200	34	8.5
	200–350	60	15.0
	>350	307	76.6
Sero-prevalence of partner	Concordant	309	77.1
	Discordant	92	22.9
Duration since diagnosis	≤2 years	131	32.7
	2.1–5 years	185	46.1
	greater than five years	85	21.2
Duration on ART	Pre ART/≤ 1 year	117	29.2
	1.1–3 years	127	31.7
	greater than three years	157	39.2
Functional radio and/or TV	Yes	313	78.1
	No	88	21.9

### Demand and unmet need for family planning

**Figure 1:**
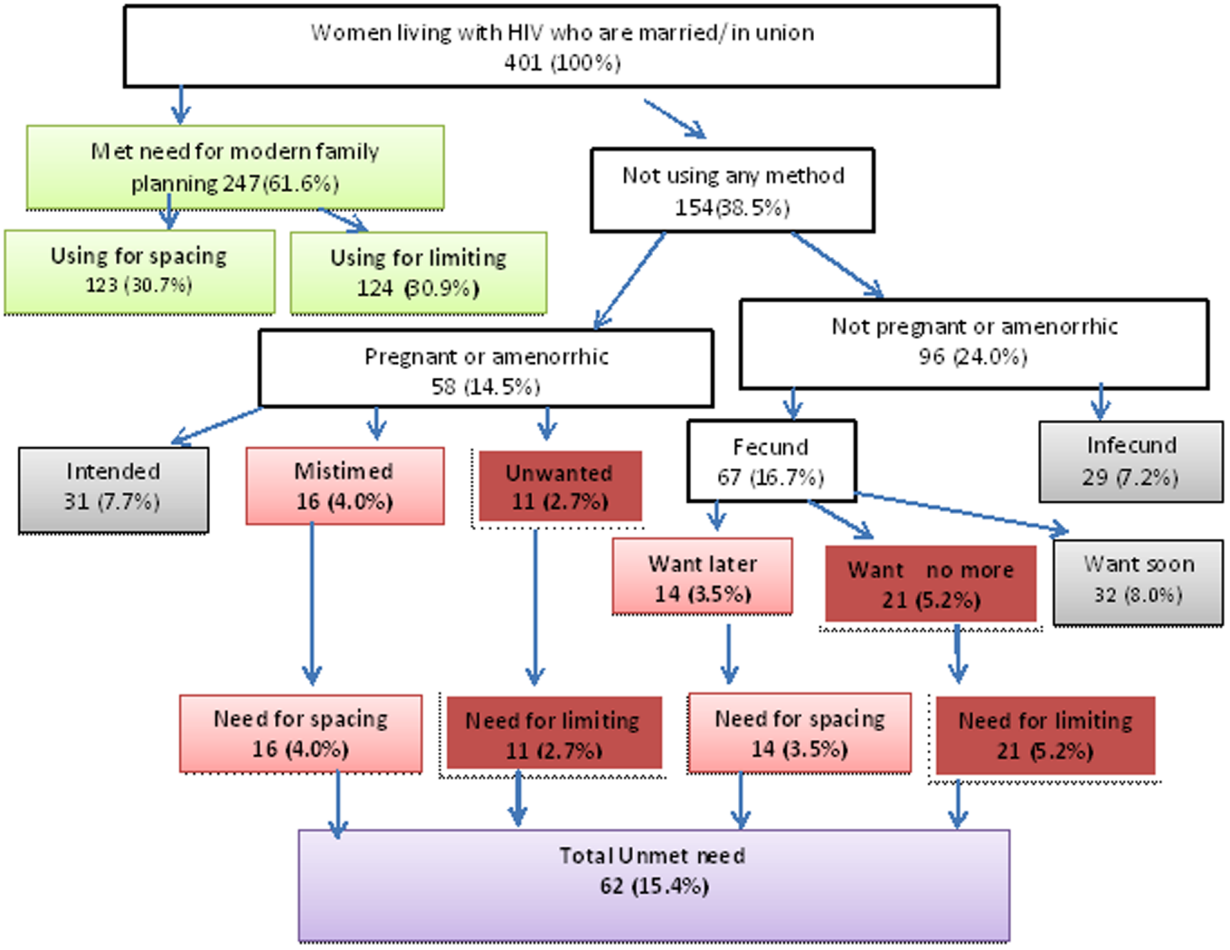
Total demand for modern family planning among married/in union women living with HIV in Western Ethiopia, March 2014.

In total, 309 participants (77%) had demand for modern family planning (38.2% for spacing and 38.8% for limiting) (Figure 1). The proportion of all respondents with met need for modern family planning was 61.6% (30.7% for spacing and 30.9% for limiting). Thus, 15.4% of respondents had unmet need for modern family planning (7.5% for spacing and 7.9% for limiting). The most common modern family planning method used was condoms plus any of the other modern family planning methods 128(31.9%), followed by injectables 101(25.2%) (Table 2).

**Table 2:** Modern family planning method utilization of married women living with HIV in western Ethiopia, March 2014.

Types of modern family planning used (n = 401)	Frequency (%)	Using for spacing (%)	Using for Limiting (%)
Condom alone	84(21.0)	50(12.50)	34(8.50)
Pills	11(2.7)	4(1.0)	7(1.7)
Injectables	101(25.2)	41(10.2)	60(15.0)
IUD	9(2.2)	4(1.0)	5(1.2)
Implants	43(10.7)	24(6.0)	19(4.7)
Tubal ligation	1(0.2)	-	1(0.2)
Condom plus any of the other modern FP	128(31.9)	55(13.7)	73(18.2)

Crude rates of family planning needs are displayed in Table 3. Women aged 35–49 had the highest proportion of unmet need for modern family planning (23.3%). Women with primary education (25(19.1%)) also had a higher prevalence of unmet need than those with higher education. Similarly, women in the lowest quantile of household income had higher unmet need (19.2%), than those in higher income quintiles.

**Table 3:** Demand for modern family planning among married women living with HIV in western Ethiopia, March 2014.

			Modern family planning			
Variables	Categories	n	Unmet	Met	demand	P value
Age	15–24	69	12(17.4)	39(56.5)	51(73.9)	**.027**
	25–34	242	29(12.0)	159(65.7)	188(77.7)	
	35–49	90	21(23.3)	49(54.4)	70(77.8)	
Education level	No formal education	171	27(15.8)	104(60.8)	131(76.6)	.462
	Primary education	131	25(19.1)	86(65.6)	111(84.7)	
	Secondary and above	99	10(10.1)	57(57.6)	67(67.7)	
Education level of partner	No formal education	91	16(17.6)	59(64.8)	75(82.4)	.309
	Primary education	124	24(19.4)	75(60.5)	99(79.8)	
	Secondary and above	186	22(11.8)	113(60.8)	135(72.6)	
Religion	Protestant Christian	163	29(17.8)	95(58.3)	124(76.1)	.439
	Orthodox Christian	186	25(13.4)	121(65.1)	146(78.5)	
	Muslim	52	8(15.4)	31(59.6)	39(75.0)	
Occupation	Daily labourer	88	17(19.3)	53(60.2)	70(79.5)	.132
	Employed- skilled	182	19(10.4)	116(63.7)	135(74.2)	
	Housewife	73	15(20.5)	41(56.2)	56(76.7)	
	Farmer	58	11(19.0)	37(63.8)	48(82.8)	
Residence	Urban	326	50(15.3)	200(61.3)	250(76.7)	.953
	Rural	75	12(16.0)	47(62.7)	59(78.7)	
Monthly income quintile	Lowest	95	15(15.8)	62(65.3)	77(81.1)	.679
	Second	82	11(13.4)	52(63.4)	63(76.8)	
	Middle	77	13(16.9)	49(63.6)	62(80.5)	
	Fourth	73	14(19.2)	38(52.1)	52(71.2)	
	Highest	74	9(12.2)	46(62.2)	55(74.3)	
Family size	2 or less	45	5(11.1)	23(51.1)	28(62.2)	.502
	3–4	195	25(12.8)	117(60.0)	142(72.8)	
	greater than four	161	32(19.9)	107(66.5)	139(86.3)	
Number of live children	No living child	47	5(10.6)	21(44.7)	26(55.3)	.582
	1–2	218	30(13.8)	136(62.4)	166(76.1)	
	>2	136	27(19.9)	90(66.2)	117(86.0)	
recent CD_4_ count	<200	34	6(17.6)	22(64.7)	28(82.4)	.748
	200–350	60	11(18.3)	35(58.3)	46(76.7)	
	>350	307	45(14.7)	190(61.9)	235(76.5)	
Sero-prevalence of partner	Concordant	309	50(16.2)	191(61.8)	241(78.0)	.573
	Discordant	92	12(13.0)	56(60.9)	68(73.9)	
Duration since diagnosis	≤2 years	131	21(16.0)	78(59.5)	99(75.6)	.911
	2.1–5 years	185	29(15.7)	116(62.7)	145(78.4)	
	greater than five years	85	12(14.1)	53(62.4)	65(76.5)	
Duration on ART	Pre ART/≤1 year	117	24(20.5)	63(53.8)	87(74.4)	.114
	1.1–3 years	127	17(13.4)	87(68.5)	104(81.9)	
	greater than three years	157	21(13.4)	97(61.8)	118(75.2)	
knowledge of MTCT	Yes	389	57(14.7)	241(62.0)	298(76.6)	**.032**
	No	12	5(41.7)	6(50.0)	11(91.7)	
Functional radio and/or TV	Yes	313	44((14.1))	194(62.0)	238(76.0)	.205
	No	88	18(20.5)	53(60.2)	71(80.7)	
Discussed about FP with partner	No	75	24(32.0)	35(46.7)	59(78.7)	**.000**
	Sometimes	42	9(21.4)	23(54.8)	32(76.2)	
	More Often	284	29(10.2)	189(66.5)	218(76.8)	
Total		401	62(15.4)	247(61.6)	309(77.0)	

In multivariable models, the following characteristics were associated with unmet need for family planning: age 25–34 years [AOR (95% CI)  = .397 (.204–.771)], not having knowledge about mother to child transmission [AOR (95% CI)  = 2.531 (1.689–9.290)], and not discussing family planning with a partner [AOR (95% CI)  = 3.616(1.869–6.996)] (Table 4).

**Table 4:** Factors associated with unmet need for modern family planning among women living with HIV in western Ethiopia, March 2014.

Variables		Unmet need for modern family planning		Crude OR (95% CI)	Adjusted OR (95% CI)
		Yes (%)	No (%)		
Age	15–24	12 (19.4)	57 (16.8)	.692 (.314–1.526)	.490 (.209–1.150)
	25–34	29 (46.8)	213 (62.8)	**.447 (.240–.835)**	**.397 (.204–.771)**
	35–49	21 (33.9)	69 (20.4)	1	1
Occupation	Daily labourer	17(27.4)	71(20.9)	1	1
	Employed- skilled	19(30.6)	163(48.1)	**.487(.239**–**.991)**	.561 (.264–1.191)
	Housewife	15(24.2)	58(17.1)	1.080(497–2.347)	.974 (.431–2.199)
	Farmer	11(17.7)	47(13.9)	.977(.421–2.271)	1.112 (.456–2.707)
Knowledge of MTCT	Yes	57 (91.9)	332 (97.9)	**1**	**1**
	No	5 (8.1)	7 (2.1)	**4.160(1.276–13.560)**	**2.531 (1.689–9.290)**
Discussed FP with a partner	No	24(38.7)	51(15.0)	**4.138 (2.229–7.683)**	**3.616(1.869–6.996)**
	rarely	9(14.5)	33(9.7)	**2.398 (1.045–5.506)**	2.305(.978–5.433)
	more often	29(46.8)	255(75.2)	1	**1**

## Discussion

This study aimed to identify demand and unmet need for modern family planning among married and in-union women living with HIV. We found that unmet need for modern family planning methods was high among HIV positive married and in-union women, reported by 15% of respondents. Our findings reinforce the fact that unmet need for family planning services among women living with HIV continues to undermine efforts to eliminate new HIV infections among children. Additional efforts to reduce unmet need and satisfy demand for family planning can eliminate HIV infections among children and reduce maternal deaths among women living with HIV in the era of better access to ART.

When compared to surveys from the general population, our reported rates of unmet need were lower than findings from Uganda [9] and Ethiopia [2]. However, when compared to studies from the HIV positive women, our results are consistent with unmet need among married women in Zimbabwe (16%) [17],higher than reports from prior demographic and health surveys in Kenya [18] but lower than findings from Uganda [19]. We also found higher rates of modern family planning (combination methods used 32%), compared to a previous report from Lesotho, which documented 6% in a similar population [20]. A possible reason for the high met need is that married women receiving HIV care have more regular contact with health care professionals. Moreover, discussions regarding family planning (emphasis on dual FP) are incorporated into the regular clinical follow-up routine of HIV patients in the study setting.

The total demand in current study was higher than findings from Ethiopia and eastern Sudan in general population [2], [21]. This may be due to perceived fear of MTCT among HIV positive women [18], [22]. Satisfying demand for modern family planning improves the prospects of mothers' survival.

We found a number of factors predicted rates of unmet need for family planning. In this study, age was one of the important factors associated with unmet need for modern family planning. Women within the age range of 25–34 were 0.45 times less likely to have unmet need for family planning than those found in the age range 35–49. This is consistent with Ethiopian DHS data from 2000 and 2005 among the general population [23]. The reason for low unmet need among women age 25–34 years is unclear, but could be related to perceived fears of being pregnant than older women and more likely to use FP. The other reason may be younger women are more likely to be literate than older [2] and more likely to discuss family planning with a partner than older women.

Those women who were not knowledgeable of MTCT were more likely to have unmet need for modern family planning compared to their counterpart. Women having knowledge of MTCT who had desire for more children may strive to get means to control birth. Therefore, efforts should be there to increase knowledge about MTCT among PLHIV and the general population.

In multivariate analysis those women who did not discuss family planning with a partner were also at significantly greater odds of unmet need for modern family planning. This is consistent with studies from Butajira, Ethiopia among women in the general population [14]. Men often play decisive roles in either supporting or hindering the use of contraceptives by their spouses. Thus, communication with a partner is vital to remove challenges such as partner opposition in fertility related decisions, including choice of modern family planning. Men should also be involved in the programmatic response in order to reduce unmet need among women living with HIV.

The primary limitation of the study was the use of cross-sectional data, which precludes assessing the causality of the associations described. There is also a risk of social desirability bias whereby women living with HIV may over-report their contraceptive use (condom use, in particular) because of perceived pressure from health workers to practice safe sex. The questionnaire was administered by nurses who were working in the clinic to minimize this bias.

## Conclusions and Recommendations

In conclusion, our study demonstrates that women living with HIV in Ethiopia have high demand for modern family planning (77%), with high rates of both unmet need (15.4%) and met need (61.6%). Younger women, poor knowledge on MTCT, and lack of discussion with partners on family planning were important factors associated with unmet need for modern FP. Efforts to address the unmet need for modern family planning and rights of women living with HIV need to be strengthened. As national programs improve availability and provision of PMTCT, policy makers and funders should not ignore the continued importance of family planning. Thus, stakeholders should act to satisfy the family planning demand focusing on knowledge of PMTCT and to promote discussion about family planning among partners.
